# Incremental cost per newly diagnosed HIV infection (NDHI): routine (RTS), targeted (TTS), and current clinical practice testing strategies (CPTS)

**DOI:** 10.7448/IAS.17.4.19606

**Published:** 2014-11-02

**Authors:** Cristina Gomez-Ayerbe, María Jesús Pérez Elías, Alfonso Muriel, Pilar Pérez Elías, Agustina Cano, Alberto Diaz, Ana Moreno, Jose Luis Casado, Cristina Santos, María Martinez-Colubi, Almudena Uranga, Fernando Dronda, Santiago Moreno

**Affiliations:** 1 Infectious Diseases, Hospital Ramón y Cajal, IRYCIS, Madrid, Spain; 2 Statistics Department, Hospital Ramón y Cajal, IRYCIS, Madrid, Spain; 3 Family Care, Centro de Salud García Noblejas, Madrid, Spain; 4 Internal Medicine, Hospital de Sanchinarro, Madrid, Spain

## Abstract

**Introduction:**

Although RTS as HIV Diagnosis was considered cost effectiveness [1], overall budget may be unaffordable for some countries. We explore Incremental cost per NDHI associated with different TS.

**Materials and Methods:**

From a health care perspective, using direct costs and Euros currency, we calculated budget and cost per NDHI of RTS (all patients were tested), TTS (Universal risk practices and clinical conditions-RP&CC - only positive were tested), and CPTS (Only patients physicians considered were tested). We considered DRIVE (Spanish acronym of HIV infection Rapid Diagnosis) study and clinical Practice outcomes. Population between 18–60 years, attending to a Hospital Emergency Room or to a Primary Care Center performed an HIV RP&CC questionnaire (Q) and an HIV rapid test (HIV RT). Unitary costs considered were: HIV RT, nurse, registry, transport and HIV confirmation when necessary, imputed to all population in RTS and CPTS and only in HIV RP&CC-Q positive in TTS analysis, while HIV RP&CC-Q costs were added to all population in TTS. Sensitivity analyses were performed with varying rates of NDHI and of positive HIV RP&CC-Q population, and different RP&CC Q sensitivity (SE) to predict HIV infection.

**Results:**

5,329 HIV RP&CC-Q and HIV RT were performed to 49.64% women, median age 37 years old, 74.9% Spaniards. In DRIVE and CP, NDHI were 4.1‰, and 1.6‰, while HIV RP&CC-Q was positive in 51.2%. HIV RP&CC-Q SE was 100%. Overall budget employed in HIV testing was in RTS 43,503€, in TTS 24,472€ and in CPTS 5,032€. Cost per 1 NDHI was 1,977€, 1,112€ and 5,032€, respectively. A reduction in cost of 865€, favouring TTS vs. RTS, while an increased cost of 824€ in CPTS vs. RTS was obtained. Considering NDHI rate of 2.6‰ saving costs increased to 1379€ in TTS, while were reduced to 576€ if NDHI rate increases 6.2‰. Effect of RP&CC-Q positivity rate was similar, if 25% saving costs were 1368€, while if 75% were reduced to 399€. Varying SE of RP&CC-Q to 95%, 91% and 50% cost saving was 810€, 754€, and 208€, and number of MHI one, two and 11.

**Conclusions:**

In DRIVE study Targeted TS with universal screening of RP&CC before an HIV rapid test is cost saving, without missing NDHI, with respect to Routine TS. Lower rates of HIV infection and RP&CC in the population, increase costs savings.
